# Successful treatment of pulmonary haemorrhage and acute respiratory distress syndrome caused by fulminant *Stenotrophomonas maltophilia* respiratory infection in a patient with acute lymphoblastic leukaemia – case report

**DOI:** 10.1186/s12879-020-05378-9

**Published:** 2020-09-10

**Authors:** Stefan Andrei, Alexandra Ghiaur, Lavinia Brezeanu, Cristina Martac, Andreea Nicolau, Daniel Coriu, Gabriela Droc

**Affiliations:** 1 1st Department of Anesthesia and Intensive Care, Fundeni Clinical Institute, 258 Soseaua Fundeni, Sector 2, 022328 Bucharest, Romania; 2 Carol Davila University of Medicine and Pharmacy, 8 Bulevardul Eroii Sanitari, 050474 Bucharest, Romania; 3 Department of Haematology, Fundeni Clinical Institute, 258 Soseaua Fundeni, Sector 2, 022328 Bucharest, Romania

**Keywords:** Stenotrophomonas maltophilia, Pulmonary haemorrhage, Acute respiratory distress syndrome

## Abstract

**Background:**

*Stenotrophomonas maltophilia*-induced pulmonary haemorrhage is considered a fatal infection among haematological patients. The outcome can be explained by the patients’ immunity status and late diagnosis and treatment.

**Case presentation:**

We present the rare case of successful outcome in a 61-year-old female who developed alveolar haemorrhage and acute respiratory distress syndrome 8 days after a chemotherapy session for her acute lymphoblastic leukaemia, in the context of secondary bone marrow aplasia. *Stenotrophomonas maltophilia* was isolated in sputum culture. The patient benefitted from early empirical treatment with colistin followed by trimethoprim/sulfamethoxazole, according to the antibiogram. Despite a severe initial clinical presentation in need of mechanical ventilation, neuromuscular blocking agents infusion, and ventilation in prone position, the patient had a favourable outcome and was discharged from intensive care after 26 days.

**Conclusions:**

*Stenotrophomonas maltophilia* severe pneumonia complicated with pulmonary haemorrhage is not always fatal in haematological patients. Empirical treatment of multidrug-resistant *Stenotrophomonas maltophilia* in an immunocompromised haematological patient presenting with hemoptysis should be taken into consideration.

## Background

*Stenotrophomonas maltophilia* is an anaerobic non-fermentative bacteria that does not cause infections in immunocompetent hosts, but might be fatal in patients with weakened immune system [1]. Several case reports and case series describing pulmonary haemorrhage caused by respiratory infection with *Stenotrophomonas maltophilia* have been reported in adult patients with haematological diseases [2]. Severe pulmonary haemorrhage due to *Stenotrophomonas maltophilia*has also been reported in neonates [3].

The literature presents a variety of cases in adults with allogeneic stem cell transplantation [4, 5] or secondary to chemotherapy-induced pancytopenia, particularly myeloid leukaemia [6, 7]. In all these cases, the mortality was very high; Mori et al. reported no survival in one review of haematological adult cases [2].

We report the case of successfully treated severe *Stenotrophomonas maltophilia* respiratory infection complicated with pulmonary haemorrhage in a chemotherapy-induced pancytopenia patient diagnosed with acute lymphoblastic leukaemia.

## Case presentation

A 61-year-old female was diagnosed in December 2018 with Philadelphia chromosome-positive acute lymphoblastic leukaemia. Her past medical history was significant for hypertension and type 2 diabetes mellitus for more than 10 years. At the moment of diagnosis, the total white blood cell count was 31 × 10^9^/L (WBC) with peripheral blood smear showing 47% blasts, haemoglobin 7.4 g/dL, and 100 × 10^9^/L platelets count (PLT). The bone marrow smears showed hypercellularity with more than 90% blast cells. The flow cytometry revealed 75% blast cells compatible with common B cell acute lymphoblastic leukaemia. The cytogenetic and fluorescence in situ hybridization analysis have identified the translocation t (9;22)(q34;q11)(Philadelphia chromosome). The molecular analysis was positive for BCR-ABL fusion gene (p210 isoform), and the BCR-ABL/ABL ratio was 31.55%. The patient received treatment according to GRAAPH2005 protocol with Imatinib 600 mg/day. The patient achieved complete morphological remission after the induction, with a molecular response BCR-ABL/ABL ratio of 0.12% IS.

The 6 months follow-up assessment showed a deep molecular response (molecular analysis ratio BCR-ABL/ABL: 0.0086% -MR4.0 log IS). The cytogenetic analysis 46,XX [20] was consistent with a complete cytogenetic response, and the bone marrow aspiration showed trilineage dysplasia. Her treatment was continued with the same regimen.

**Fig. 1:**
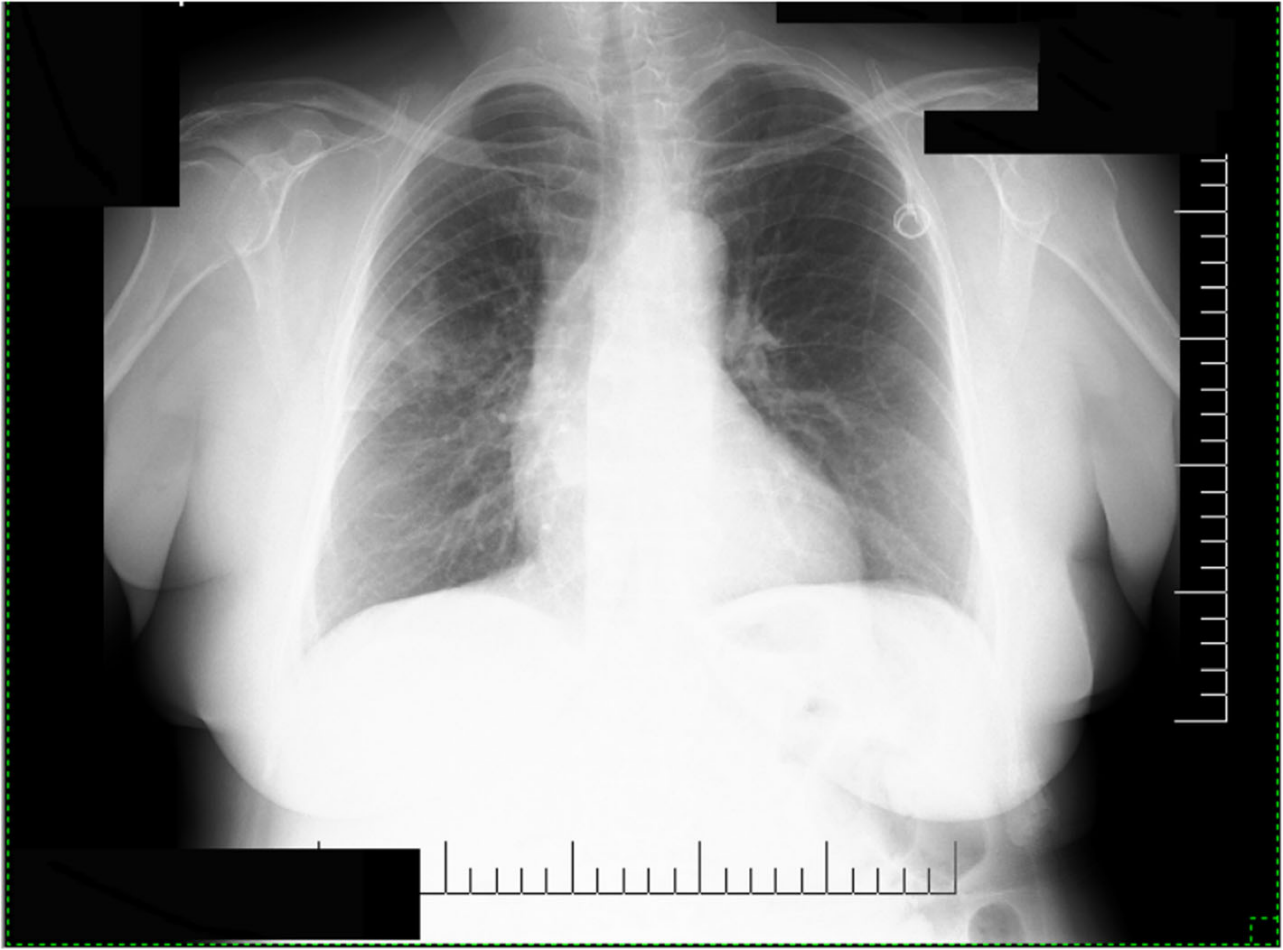
Chest radiography showed a small area of right perihilar infiltration (day 8 after chemotherapy)

On day eight after consolidation therapy with cycle 3 (Methotrexate 1 g/m^2^ at day 1, Cytarabine 500 mg/m^2^ BID at day 2 and day 3), a severe aplasia developed (0.76 × 10^9^/L WBC, 0 × 10^9^/L absolute neutrophil count (ANC), 7 × 10^9^/L PLT), and she became febrile with a mild cough and a small area of right perihilar infiltration on chest X-rays (Fig. 1), despite prophylactic treatment with levofloxacin. Sputum culture and peripheral blood cultures were performed, and the patient was started on empirical broad-spectrum antibiotics (piperacillin-tazobactam, amikacin) and fluconazole.

Over the next 2 days, the respiratory status worsened, and the patient developed severe cough and hemoptysis, dyspnea and an increased in the oxygen demand. Considering the worsening clinical status, the risk of multidrug-resistant (MDR) germs infection, as well as severe bone marrow aplasia with multiple hospitalizations, the antibiotic treatment was escalated to meropenem, linezolid, amikacin, and fluconazole was switched to voriconazole. The patient received platelets and subcutaneous granulocyte colony-stimulating factor treatment.

**Fig. 2:**
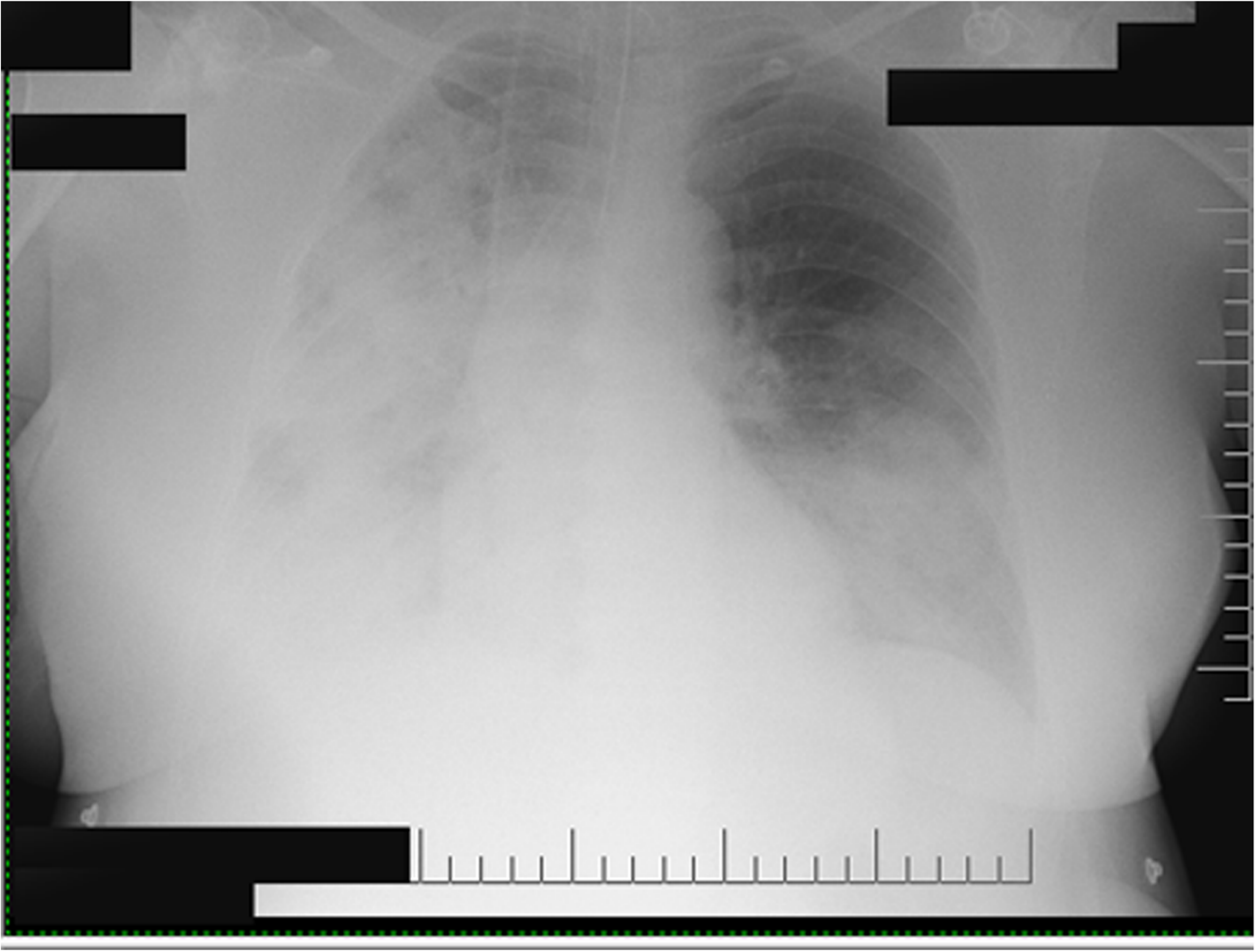
Chest X-rays at the moment of ICU admission, after tracheal intubation and mechanical ventilation initiation. Alveolar consolidation of the entire right lung and the lower lobe of the left lung are seen

Four days after the onset of fever (11 days after chemotherapy), the patient was admitted to ICU for type I respiratory failure and pulmonary haemorrhage. On ICU admission, the patient was conscious and oriented, very dyspneic and tachypneic with a respiratory rate of 38/min, and a SpO_2_78% on 12 l per minute (lpm) O_2_ on facial mask, and bilateral auscultatory pulmonary crackles. The blood pressure was 200/120 mmHg, and the heart rate was 100 beats per minute, sinus rhythm. There were no signs of peripheral hypoperfusion. The temperature was 37.3^o^ Celsius. The arterial blood gases revealed a pH 7.39, PaO_2_64 mmHg, PaCO_2_ 30 mmHg, lactate 1.5 mmol/L. The antibiotic cover was broadened with the addition of colistin. After 2 h on high flow nasal oxygen (FiO_2_ 1, flow 50 lpm) and in the absence of clinical improvement (persistent respiratory effort), mechanical ventilation (6 ml/kg, positive end-expiratory pressure titrated at 12 cm H_2_O, plateau pressure 28 cm H_2_O), sedation, and continuous muscle paralysis were initiated. One-hour post intubation, the PaO_2_/FiO_2_ ratio was to 52, on 100% O_2_. The decision to initiate ventilation in prone position for 18 h was made at this point, with poor respiratory responsiveness (PaO_2_/FiO_2_ ratio 114). The chest X-rays at this moment is shown in Fig. 2. The acute respiratory distress syndrome (ARDS) diagnosis was established. Within the next 24 h in the ICU (day 12 after chemotherapy), the patient recovered from aplasia (10.14 × 10^9^/L WBC, 8.36 × 10^9^/L ANC, 103 × 10^9^/L PLT). The neuromuscular blockade was discontinued after 48 h.

**Fig. 3:**
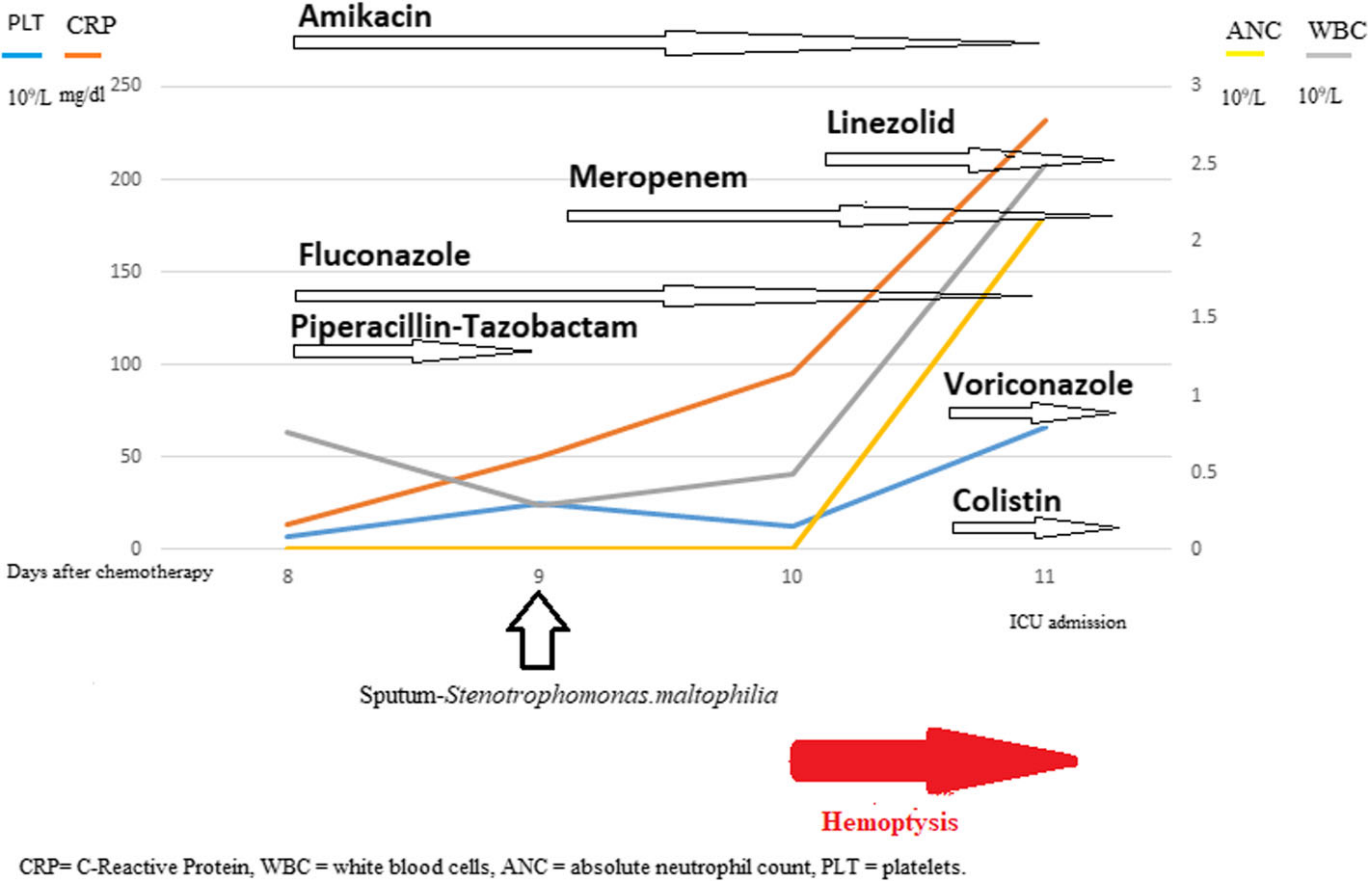
Summary of the clinical and biochemical parameters before ICU admission

The clinical state and blood results prior to ICU admission are summarized in Fig. 3.

The sputum culture was positive for *Stenotrophomonas maltophilia* (cultured on day 9 after chemotherapy, 2 days before the ICU admission). Therefore, the antibiotic treatment has been de-escalated to colistin (4.5 MIU, BID, after initial bolus dose of 9 MIU) and trimethoprim/sulfamethoxazole (800 mg/160 mg, QID). After other 2 days, the antibiogram showed a germ susceptible to trimethoprim/sulfamethoxazole (TMP/SMX), intermediary susceptible to levofloxacin, and resistant to all beta-lactams and aminoglycosides (no determined MIC available). The antibiotic treatment has been further de-escalated the next day to TMP/SMX only for a total duration of 7 days. Unfortunately, the laboratory was logistically unable to test the susceptibility to colistin at this point. All blood cultures were reported negative during the hospital stay.

The respiratory improvement allowed for a bronchoscopy with bronchoalveolar lavage, 3 days after ICU admission, which showed a non-haemorrhagic mucosa, and numerous blood clots. The cytologic examination was very rich in red blood cells with some macrophages, rare lymphocytes and neutrophils. Respiratory cultures from the bronchoalveolar lavage were negative.

**Fig. 4:**
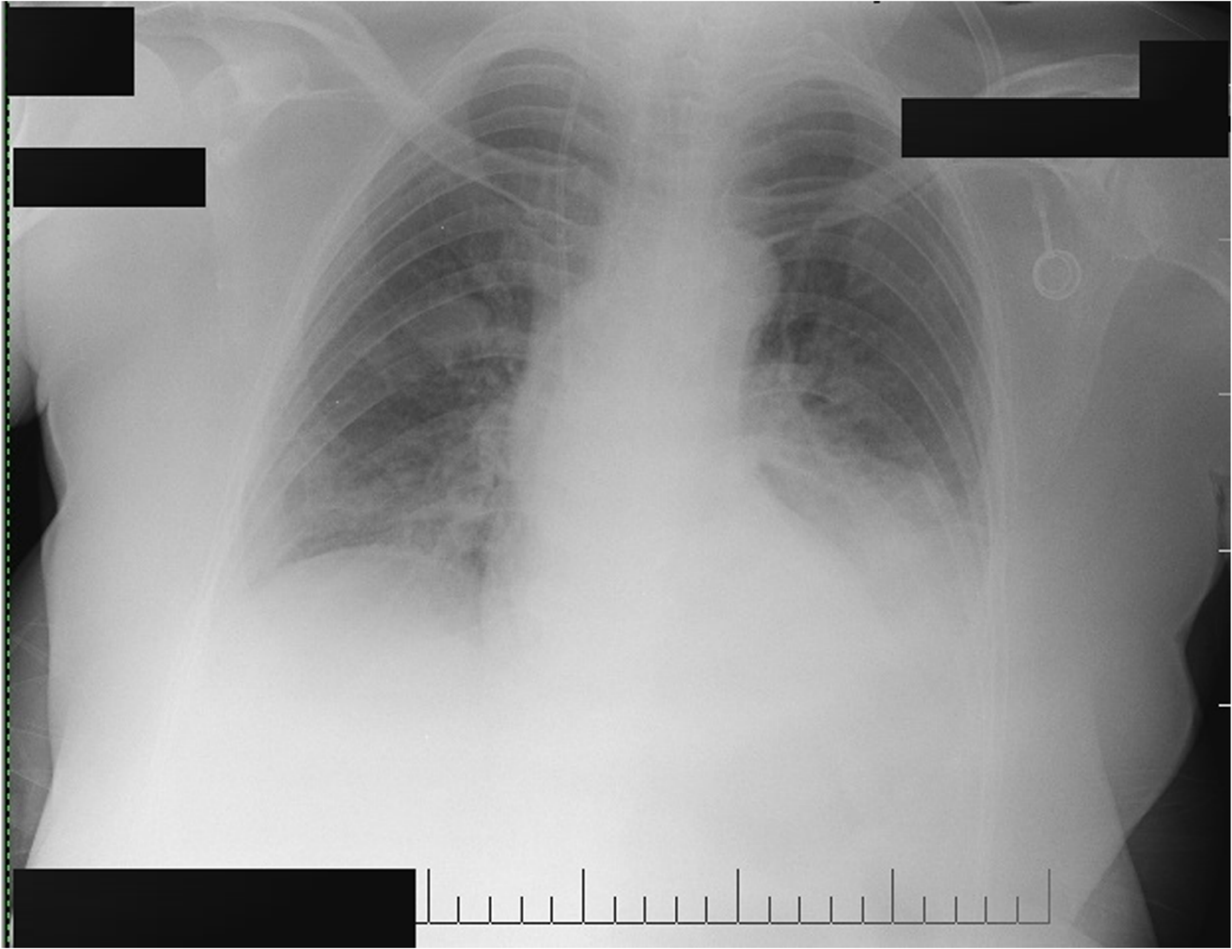
Chest X-rays after extubation (day 20 of ICU). The regression of the bilateral infiltrates and mild bilateral pleural effusion can be noticed

The clinical course was marked by a failed extubation at day 11 of ICU, explained by ICU-induced neuromyopathy and ventilator-associated pneumonia (VAP) with MDR *Pseudomonas aeruginosa* susceptible only to colistin (MIC 2), and resistant to carbapenems and other antibiotics. Treatment with IV and nebulized colistin was introduced for a total duration of 2 weeks. The respiratory status progressively improved, allowing the successful extubation on day 20 in ICU. The chest X-rays post extubation is shown in Fig. 4.

The patient was transferred to the haematology ward after 26 days of ICU stay and 37 days after chemotherapy. The patient was discharged home soon after, on family request, and her clinical condition and respiratory function were excellent at 10 months follow up.

## Discussion and conclusion

We reported a case of severe acute respiratory distress syndrome (ARDS) with alveolar haemorrhage and a good response to treatment. To our knowledge, this is the first reported case of pulmonary haemorrhage and ARDS caused by a fulminant *Stenotrophomonas maltophilia* respiratory infection in Eastern Europe, and it seems to be a rare case of positive outcome in a patient with haematological malignancy.

The patients diagnosed with acute leukaemia have a high risk of infection with opportunistic pathogens. The pulmonary haemorrhage caused by *Stenotrophomonas maltophilia* is a rare condition with a poor outcome. Several reports have shown the presence of predisposing factors like severe thrombocytopenia, severe and prolonged neutropenia, the previous use of quinolones, corticosteroids, and immunosuppressive therapy [2, 8, 9]. Our patient had grade IV thrombocytopenia and neutropenia secondary to chemotherapy. She also had a history of prolonged hospitalization for chemotherapy and complications related to chemotherapy, and she was treated with multiple classes of antibiotics, including quinolones, and she had prolonged corticosteroids use.

In the published literature, we found 34 haematological patients with *Stenotrophomonas maltophilia* and pulmonary haemorrhage [2, 8]. Most patients (22 out of 34) were diagnosed with acute myeloid leukaemia, and the mortality rate was 100%, and the survival length was 0–43 days. It is important to notice the high early death rate: almost 80% of patients died in the first 3 days after the onset of the respiratory symptoms. Another significant point is that 37% of patients had a non-remission state of their haematological disease.

The particularity of our case is that the patient recovered very soon from severe aplasia after ICU admission (day 1) permitting the respiratory stabilization in the absence of recurrent hemoptysis. Also, we speculate that the empirically introduced colistin at the moment of ICU admission for ARDS, associated with neutrophil count recovery, might have been efficient in stopping the bacterial proliferation. The only available options to treat the *Stenotrophomonas maltophilia* infection*,* in this case, were TMP/SMX and colistin - which has not been tested because of laboratory unavailability of the antibiogram kit. The literature-reported resistance of *Stenotrophomonas maltophilia* to colistin was < 10% [10]. There is no standard therapy for the treatment of severe *Stenotrophomonas maltophilia* pneumonia with pulmonary haemorrhage, but combination antibiotic therapy represents an alternative to consider in critical situations [9]. In our case, the patient received colistin and TMP/SMX combination for 2 days, the time between the germ identification and the antibiogram results, followed by further de-escalation to TMP/SMX only. The decision to continue IV colistin has been taken considering the fact that the patient has been already exposed to levofloxacin, the possibility of a MDR germ and the unknown bioavailability of enterally administered TMP/SMX in a severely ill patient with reactive digestive ileus and gastric stasis (the IV form is not available in our country).

Some retrospective studies have shown that, during ICU stay, the absence of neutropenia recovery and the presence of organ failure are associated with poor outcome in the critically ill patient with malignancy [11, 12]. Furthermore, according to other reports, survival was higher for patients who underwent a first-line chemotherapy, had lobar ARDS and who received antibiotic treatment active on difficult-to-treat bacteria like *Pseudomonas aeruginosa* and *Stenotrophomonas maltophilia* [11]. The duration of neutropenia seems to be correlated with short-term mortality, while 30-day mortality is affected by organ dysfunction [12, 13].

One explanation for the high mortality in this clinical situation could be that the patients do not survive the first days of ICU because of the difficulty of proper pathogen isolation and its corresponding antibiogram, allowing targeted antibiotic treatment [2]. Indeed, 3 days were needed to identify the pathogen in the respiratory samples, and 5 days to have complete cultures and susceptibilities.

The consequence of initial wide spectrum antibiotic treatment was the development of a late VAP with MDR *Pseudomonas aeruginosa.* We are not able to provide a full explanation for this pneumonia with a MDR germ susceptible to colistin in a patient who already received colistin, as a serum colistin level was not measured (logistically unavailable). Nevertheless, we may speculate that this might have happened due to changes in the volume of distribution in the patient with prolonged ICU hospitalization. The efficacy of associated nebulized colistin might be an argument for an increased active concentration in the targeted organ. Overall, the prolonged treatment with colistin was well tolerated with no neurotoxicity, nor nephrotoxicity.

The clinical management of this case had limitations and debatable aspects, such as the unavailable quantitative sputum culture, a long delay in obtaining germ identification and antibiogram, the unavailable colistin-resistance kit for *Stenotrophomonas maltophilia*, the unavailable IV form for TMP/SMX, or the impossibility to determine the colistin serum levels. The rapid escalation in the antibiotic cover might be partially explained by our national known struggle and local experience with bacterial resistance.

Infections with multi-resistant opportunistic pathogens in haematological patients treated with chemotherapy is a complication that associates therapeutic challenges and high mortality rate. This clinical case highlights the severity and rapid progression of *Stenotrophomona smaltophilia* pneumonia in a patient with acute lymphoblastic leukaemia. The patient had a good outcome following treatment with Colistin and TMP/SMX, associated with rapid recovery cell count after the pneumonia onset.

This case might be an argument that the clinician might consider the empirical covering of *Stenotrophomonas maltophilia*, particularly in the immunocompromised haematological patient presenting with hemoptysis, as it proves that *Stenotrophomonas maltophilia* is not always lethal in fragile haematological patients.

## Data Availability

Supplementary medical data are available at request.

## Abbreviations

ABL: Abelson murine leukemia viral oncogene homolog 1
ANC: Absolute neutrophil count
ARDS: Acute respiratory distress syndrome
BCR: Breakpoint cluster region protein
FiO_2_: Fraction of inspiratory oxygen
Hb: Hemoglobin serum level
Lpm: Liter per minute
MDR: Multidrug resistant
MIU: Millions international units
PaO_2_: Arterial oxygen pressure
PLT: Platelets count
TMP/SMX: Trimethoprim/sulfamethoxazole
WBC: White blood cells count
VAP: Ventilator-associated pneumonia
